# The Arrangement of the Blood-Vessels in the Mucous Membrane of the Stomach

**Published:** 1850-12

**Authors:** 


### ANATOMY AND PHYSIOLOGY.

*The Arrangement of the Blood-Vessels in the Mucous Membrane of the Stomach.*—The course of the vessels, as far as the point where they pierce the walls of the stomach to pass to their final distribution, is well-known. Their minute distribution has never been perfectly described. It has lately been carefully examined by Frey, in men, dogs, cats, sheep, and guinea-pigs.

1. The veins pass usually obliquely through the muscular coat, and reach the submucous tissue ; they bend here into a longitudinal direction, and run at a little distance below the blind extremities of the tubes. They are of large and regular diameter, and frequently anastomose with each other. Frey calls them "basal veins." From these basal veins arise, at right angles, numerous branches, which run in a straight or gently-waved direction to the free surface of the mucous membrane.

When they have passed half way through the membrane, they sometimes divide into two branches, which continue in the same course as the parent vessel would have taken. At the free surface of the mucous membrane, these vertical veins give off many branches, which form a net-work. When this occurs there is a most important and sudden diminution in the size of the veins. The basal veins have a diameter from 1-10 to 1-20 of an inch; the vertical branches vary from 1-18 to 1-48; the veins of capillary net-work vary from 1-70 to 1-300.

2. The arteries, like the veins, penetrate the muscular coat obliquely and pass with the veins till these latter bend to form the "basal veins." They are much smaller than the veins,—not more than one-third or one-fourth of their diameter. They form no vessels corresponding to the basal veins, but divide at once into branches, which freely anastomose; from this net-work branches arise, which pass towards the surface; when they reach the tubes, they pass longitudinally between these, communicating by short crossbranches, coming off at sharp angles; these sometimes form a ring round the tubes, but if a ring is formed at this point, it is irregular and exceptional. When the arteries reach the mouths of the tubes, they divide to form a second net-work, and these branches form rings round each tube, or, more seldom, a ring round two adjoining tubes. From these circles the capillaries pass into the veins.

In this arrangement, (the description of which we have condensed as much as possible,) Frey sees two points very worthy of attention: one is, the sudden enlargement of the veins, so soon below their capillary net-work, which seems especially calculated to promote absorption; the other point is the arterial arrangement, which Frey compares to that of the glomeruli of the kidney, as unfolded by the beautiful researches of Bowman.—*London Med. Times, from Henle's Zeitschrift.*

